# Targeting GM-CSF in COVID-19 Pneumonia: Rationale and Strategies

**DOI:** 10.3389/fimmu.2020.01625

**Published:** 2020-07-03

**Authors:** Aldo Bonaventura, Alessandra Vecchié, Tisha S. Wang, Elinor Lee, Paul C. Cremer, Brenna Carey, Prabalini Rajendram, Kristin M. Hudock, Leslie Korbee, Benjamin W. Van Tassell, Lorenzo Dagna, Antonio Abbate

**Affiliations:** ^1^Wright Center for Clinical and Translational Research, Virginia Commonwealth University, Richmond, VA, United States; ^2^First Clinic of Internal Medicine, Department of Internal Medicine, University of Genoa, Genoa, Italy; ^3^Pauley Heart Center, Division of Cardiology, Department of Internal Medicine, Virginia Commonwealth University, Richmond, VA, United States; ^4^Division of Pulmonary, Critical Care, and Sleep Medicine, Department of Medicine, UCLA David Geffen School of Medicine, Los Angeles, CA, United States; ^5^Heart and Vascular Institute, Cleveland Clinic, Cleveland, OH, United States; ^6^Cincinnati Children's Hospital Medical Center, Cincinnati, OH, United States; ^7^Respiratory Institute, Cleveland Clinic, Clevaland, OH, United States; ^8^Division of Pulmonary, Critical Care and Sleep Medicine, University of Cincinnati, Cincinnati, OH, United States; ^9^Division of Pulmonary Biology, Cincinnati Children's Hospital Medical Center, Cincinnati, OH, United States; ^10^Academic Regulatory & Monitoring Services, LLC, Cincinnati, OH, United States; ^11^Unit of Immunology, Rheumatology, Allergy and Rare Diseases, IRCCS San Raffaele Scientific Institute and Vita-Salute San Raffaele University, Milan, Italy

**Keywords:** COVID-19, GM-CSF, IL-6, mavrilimumab, cytokine release syndrome, SARS-CoV-2

## Abstract

COVID-19 is a clinical syndrome ranging from mild symptoms to severe pneumonia that often leads to respiratory failure, need for mechanical ventilation, and death. Most of the lung damage is driven by a surge in inflammatory cytokines [interleukin-6, interferon-γ, and granulocyte-monocyte stimulating factor (GM-CSF)]. Blunting this hyperinflammation with immunomodulation may lead to clinical improvement. GM-CSF is produced by many cells, including macrophages and T-cells. GM-CSF-derived signals are involved in differentiation of macrophages, including alveolar macrophages (AMs). In animal models of respiratory infections, the intranasal administration of GM-CSF increased the proliferation of AMs and improved outcomes. Increased levels of GM-CSF have been recently described in patients with COVID-19 compared to healthy controls. While GM-CSF might be beneficial in some circumstances as an appropriate response, in this case the inflammatory response is maladaptive by virtue of being later and disproportionate. The inhibition of GM-CSF signaling may be beneficial in improving the hyperinflammation-related lung damage in the most severe cases of COVID-19. This blockade can be achieved through antagonism of the GM-CSF receptor or the direct binding of circulating GM-CSF. Initial findings from patients with COVID-19 treated with a single intravenous dose of mavrilimumab, a monoclonal antibody binding GM-CSF receptor α, showed oxygenation improvement and shorter hospitalization. Prospective, randomized, placebo-controlled trials are ongoing. Anti-GM-CSF monoclonal antibodies, TJ003234 and gimsilumab, will be tested in clinical trials in patients with COVID-19, while lenzilumab received FDA approval for compassionate use. These trials will help inform whether blunting the inflammatory signaling provided by the GM-CSF axis in COVID-19 is beneficial.

## Introduction

Coronavirus disease 2019 (COVID-19) is caused by severe acute respiratory syndrome coronavirus 2 (SARS-CoV-2) with a clinical spectrum ranging from asymptomatic/pauci-symptomatic forms to severe pneumonia leading to respiratory failure, need for mechanical ventilation, and death (1). To date, no specific treatment is approved for COVID-19, and management is supportive. Severe COVID-19 pneumonia seems to be mediated by a cytokine storm (2, 3). Therefore, therapies that target hyperinflammation may be effective.

## Role of IL-1β and IL-6 in Hyperinflammation in COVID-19

In a recent report, patients with COVID-19 needing intensive care unit (ICU) admission showed a cytokine profile similar to that of secondary hemophagocytic lymphohistiocytosis with increased levels of several inflammatory cytokines [interleukin (IL)-2, IL-7, granulocyte-colony stimulating factor (G-CSF), granulocyte-monocyte stimulating factor (GM-CSF), interferon-γ-inducible protein 10 (IP-10), monocyte chemoattractant protein 1 (MCP-1), macrophage inflammatory protein 1-α (MIP1-α), and tumor necrosis factor-α (TNF-α)] (4). Additionally, increased levels of ferritin and IL-6 have been shown to correlate with a worse prognosis [(4–11); Supplementary Table 1]. These observations underline that COVID-19 is a complex disease capable to combine different patterns of inflammatory biomarkers. Indeed, most of the infections can trigger the release of IL-1β from the inflammasome (12) followed by the production of IL-6 that increases the circulating levels of C-reactive protein, the prototypical acute-phase reactant (13). In viral infections, including COVID-19, elevated levels of the pro-inflammatory cytokine IL-18, that derives from the inflammasome as IL-1β, are found along with high levels of ferritin (13), thus replicating the events commonly observed in the macrophage activation syndrome (14). Altogether, these findings support the hypothesis that a maladaptive hyperinflammatory response to the virus orchestrated by IL-6, IL-1β, and eventually GM-CSF—referred to as cytokine storm—rather than the virus itself may drive the lung damage leading to hypoxia and acute respiratory failure. Immunomodulation may be beneficial in the treatment of hyperinflammation-associated conditions.

Data supporting the role of hyperinflammation in sepsis-related acute respiratory distress syndrome (ARDS) are derived from a sub-group analysis of a phase 3 randomized controlled trial of IL-1 receptor antagonist (anakinra), which showed significant survival benefit in patients treated with anakinra compared to placebo (15). IL-1β is an upstream pro-inflammatory cytokine that is released following activation of the inflammasome in response to infection and/or injury (16).

IL-6 is a pleiotropic cytokine that influences several processes, such as acute-phase protein generation, inflammation, and antigen-specific immune responses (17). In the innate immune response, IL-6 is produced by myeloid cells [e.g., macrophages and dendritic cells (DCs)] following the recognition of sterile or non-sterile stimuli through toll-like receptors at the site of infection or tissue injury. In the adaptive immune response, IL-6 is a critical modulator of plasma B-cell differentiation and antibody production (18). A deregulated IL-6 expression is involved in the pathogenesis of several disorders, such as chronic inflammatory diseases, autoimmune diseases, and tumor development (19, 20). Cytokine release syndrome (CRS) represents an on-target effect of chimeric antigen receptor (CAR) T-cell therapy and consists of a systemic inflammatory response due to a massive cytokine release, including IL-6, GM-CSF, and interferon-γ, following the *in vivo* activation of CAR T-cells (21, 22). The incidence of CRS after CAR T-cell therapy ranges from 50 to 100% with 13–48% of patients having severe CRS (23). Tocilizumab, an IL-6 receptor blocker, has been approved for the treatment of severe CRS after CAR T-cell therapy in light of its association with a rapid improvement of clinical manifestations and a decrease in the aforementioned cytokines along with a low toxicity for CAR T-cells (18).

Different trials are recruiting patients with COVID-19 pneumonia to test whether IL-6 receptor blockers (tocilizumab, sirukumab, and sarilumab: ChiCTR2000029765, NCT04306705, NCT04315480, NCT04317092; NCT04315298, NCT04322773, and NCT04321993) and an IL-1 receptor blocker (anakinra, NCT04324021, NCT04364009, NCT04412291, NCT04366232, NCT04357366, NCT04341584, NCT04339712, and NCT04362943) improve COVID-19 pneumonia outcomes. The identification and treatment of hyperinflammation using existing therapies with understood safety profiles that are either in clinical development or approved for other indications represent a valid option to cope with the immediate need to reduce the rising mortality of COVID-19.

## GM-CSF: a Key Mediator of Inflammation and Injury

**Figure 1:**
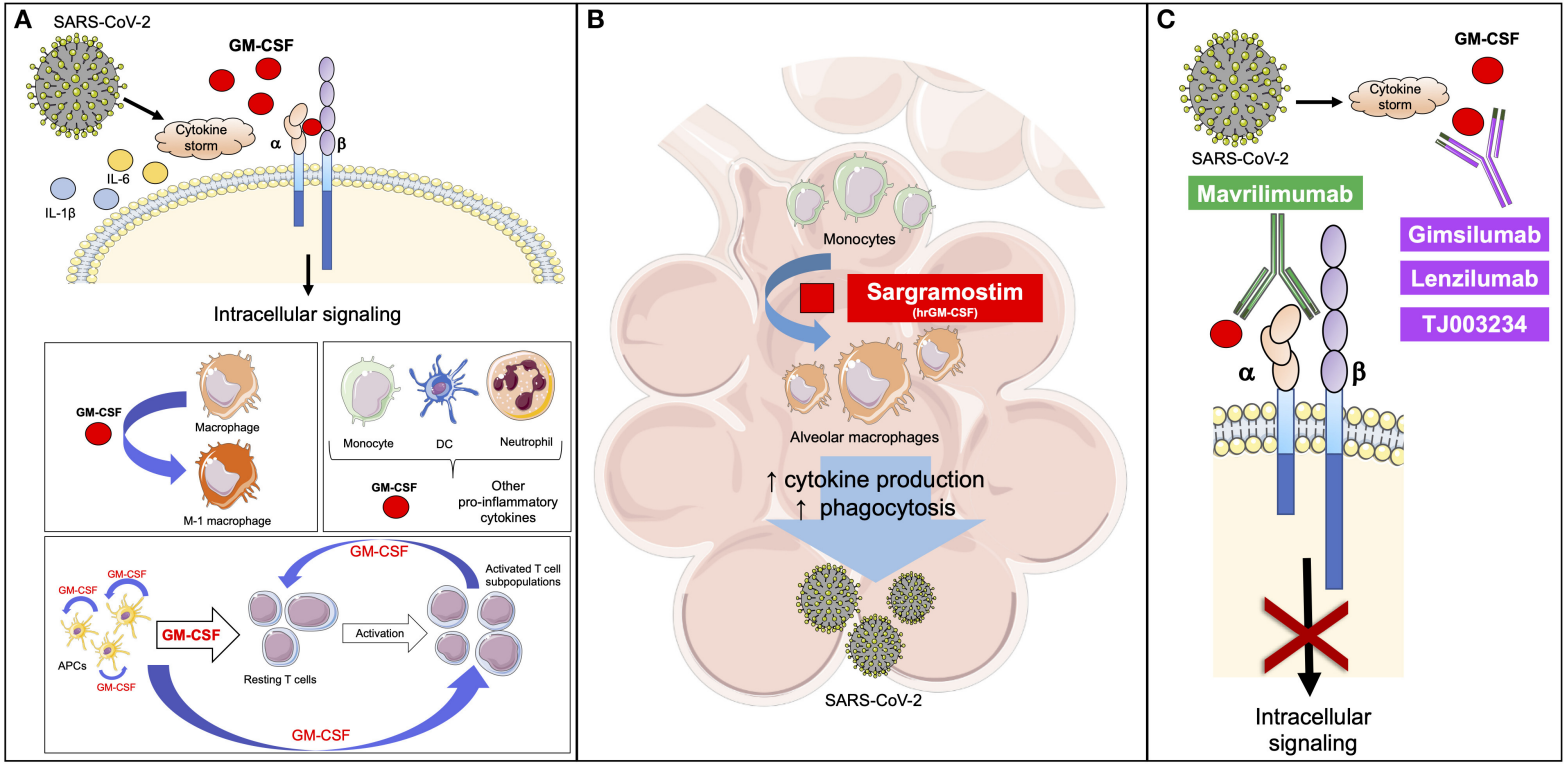
GM-CSF is involved in the response to SARS-CoV-2. **(A)** SARS-CoV-2 induces a cytokine storm with increased levels of inflammatory mediators, including GM-CSF. GM-CSF binds the α-chain of GM-CSF receptor, while the β-chain transduces the intracellular signaling. GM-CSF promotes the polarization of macrophages to the M-1 phenotype and stimulates the activation of myeloid cells that release inflammatory cytokines, like GM-CSF. APCs release GM-CSF to stimulate the differentiation of resting T cells to active T cell subpopulations. APC-derived GM-CSF promotes further release of GM-CSF through an autocrine signal. T cell-derived GM-CSF is critical to maintain T cell functions and enhance APC activity. **(B)** GM-CSF is involved in the differentiation of alveolar macrophages, thus enhancing the clearance of respiratory microbes through an increase in phagocytosis and release of pro-inflammatory cytokines (IL-1β, IL-6, and TNF-α) in a feed-forward inflammatory loop. Based on previous experiences, the early administration of a rhGM-CSF, like sargramostim, may improve the initial response against viruses, including SARS-CoV-2. **(C)** Mavrilimumab prevents GM-CSF from binding to the α-chain of its receptor, while gimsilumab, lenzilumab, and TJ003234 directly bind GM-CSF with the final common result of blocking the intracellular signaling. Based on the current knowledge, these agents can be used to reduce the hyperinflammation caused by SARS-CoV-2 in the course of the disease. Differently from rh-GM-CSF, these agents should be considered later in order to not negatively impact the favorable effects of GM-CSF on the immune response. APC, antigen presenting cell; DC, dendritic cell; GM-CSF, granulocyte-macrophage colony-stimulating factor; SARS-CoV-2, severe acute respiratory syndrome coronavirus 2. This figure has been partially created using Servier Medical Art templates, which are licensed under a Creative Commons Attribution 3.0 Unported License; https://smart.servier.com.

In an attempt to approach hyperinflammation upstream of both IL-1 and IL-6 and to target neutrophils as well as macrophages, GM-CSF may be considered as an appealing mediator. GM-CSF is generally perceived as a pro-inflammatory cytokine and is produced by many cells, including macrophages, T-cells, fibroblasts, endothelial cells, epithelial cells, and tumor cells (24), with most of the production occurring at sites of inflammation (25). GM-CSF signals are mediated by the GM-CSF receptor (GM-CSF-R) consisting of a specific ligand-binding α-chain (GM CSF-Rα) and a signal-transducing β-chain (GM CSF-Rβ) (Figure 1A). Downstream signaling of GM-CSF-R includes Janus kinase 2 (JAK2)/signal transducer and activator of transcription 5 (STAT5), nuclear factor kappa-light-chain-enhancer of activated B cells (NF-κB), extracellular signal-regulated kinase (ERK), and the phosphoinositide 3-kinase (PI3K)-Akt pathway (26–29). Importantly, ERK activity is responsible for GM-CSF-mediated human monocyte survival *in vitro* (27). Interferon regulatory factor 4 (IRF4) is a hemopoietic-specific transcription factor that has been involved in the induction of DC-like properties in monocytes treated with GM-CSF (30, 31). Recently, Achuthan et al. found that GM-CSF is capable to up-regulate IRF4 expression via Jumonji domain-containing protein D3 (JMJD3) demethylase in monocytes/macrophages (32). Increased levels of IRF4 are responsible for the production of chemokine (C-C motif) ligand 17 (CCL7), which is involved in inflammation and tissue remodeling, as occurs in arthritis (29). The GM-CSF-IRF4 signaling was also described to up-regulate major histocompatibility complex (MHC) class II expression in mouse bone marrow cultures and macrophages (33, 34).

GM-CSF levels are low or undetectable in normal conditions; however, any immune trigger can rapidly increase concentrations, as it has been seen in the lungs of patients with asthma or within the synovial fluid of patients with arthritis (35). Bacterial endotoxins and inflammatory cytokines (e.g., IL-1β, IL-6, and TNF-α) potently induce GM-CSF (25). Indeed, increased mRNA expression for TNF-α, IL-1β, and IL-6 were reported in monocytes/macrophages treated with GM-CSF (32). These findings led to hypothesize that GM-CSF is part of the inflammatory milieu of some inflammatory/autoimmune reactions. GM-CSF would work as a co-regulator along with TNF-α, IL-6, and IL-1, as part of a positive feed-forward inflammatory loop involving monocytes/macrophages, fibroblasts, and endothelial cells (36–38), but also DCs and Th cells (39–41). IL-6, however, was found to induce intestinal and splenic production of GM-CSF (42), thus promoting systemic effects, like an increase in splenic macrophage precursors. The importance of IL-1β and the IL-1 receptor/myeloid differentiation primary response (MyD88) signaling axis appears of importance in the regulation of GM-CSF by CD4^+^ and γδ T cells (43). IL-1β, together with TNF-α, can also promote monocyte viability via GM-CSF while not inducing any specific macrophage polarization (44).

Increased levels of GM-CSF have been found in the bronchoalveolar fluid of patients with ARDS compared with healthy controls (45, 46). Higher levels were observed in the early phases (1–3 days) with a progressive decrease in late stages (day 14) (46). GM-CSF may indirectly contribute to ARDS by the suppression of neutrophil apoptosis (45, 46) as activated neutrophils play a major role in the microvascular damage contributing to lung damage (47, 48).

Limited evidence describes a regulatory role for GM-CSF through the promotion of DC differentiation to a tolerogenic profile, thus increasing the number and function of regulatory T-cells (49). This can also lead to T-cell hypo-responsiveness and/or anergy (50). The mechanisms underlying pro-inflammatory and immunomodulatory phenotypes of GM-CSF are not fully understood and need to be further investigated. These properties are hypothesized to depend on the dose and the presence of other cytokines in the setting of the immune response. At lower doses, GM-CSF stimulates the tolerogenesis of myeloid cells involved in the regulatory T-cell homeostasis (49), while at higher doses GM-CSF causes myeloproliferation, leading to a sustained immune response (51).

GM-CSF-derived signals are critically involved in the differentiation of macrophages and in the proliferation and activation of other immune cells. Alveolar macrophages (AMs) are essential to clear respiratory microbes (52, 53), and their depletion has been associated with increased disease severity in murine models of influenza infection (54, 55). Therefore, several pre-clinical studies reported that the intranasal administration of GM-CSF prior to inducing an experimental viral infection conferred resistance to respiratory pathogens through an increased proliferation of AMs [(56, 57); Figure 1B]. This is probably due to an enhanced clearance of the virus, thus limiting the direct damage provided by the virus itself. Recently, a subset of AMs, the nerve-associated interstitial alveolar macrophages (NAMs), have been identified and characterized in human and murine lung (58). NAMs seem to originate from the yolk sac and, differently from the other AMs, require colony-stimulating factor 1 (CSF1) and not GM-CSF for development and maintenance in adulthood. Mouse models of influenza virus infection on selectively NAM-depleted animals suggest a central role for NAMs in the negative regulation of virus-induced inflammation, whereas the other GM-CSF-dependent AMs display a pro-inflammatory profile (58). In addition, GM-CSF receptor activation triggers stimulation of multiple downstream signaling pathways, including JAK2/STAT5, the mitogen-activated protein kinase (MAPK), and the PI3K, all fundamental in activation and differentiation of myeloid cells [(25, 37); Figure 1A].

Along with its key role in inflammation, GM-CSF is critical in lung physiology. This has been clearly highlighted by GM-CSF-deficient and GM-CSF receptor-deficient mice which develop pulmonary alveolar proteinosis (PAP) because AMs require GM-CSF to differentiate (59). The poor differentiation of these macrophages is responsible for the accumulation of surfactant proteins, saturated phosphatidylcholine, and cholesterol, leading to PAP. Indeed, local expression of GM-CSF in the lung is able to restore normal surfactant homeostasis and clearance in the setting of PAP (60). Additionally, GM-CSF-deficient mice show a persistent, low-grade inflammation resulting from inappropriate responses to commensal microbes. This chronic inflammation predisposes mice to develop different kinds of tumors (61). To date, no function-altering GM-CSF mutations have been identified in humans. However, an autoimmune form of PAP can develop in humans and is associated with high levels of neutralizing GM-CSF autoantibodies that inhibit GM-CSF signaling (62). A congenital form of PAP ending up with a complete inhibition of the macrophage clearance of surfactant has also been described and is caused by mutations in *CSF2RA* or *CSF2RB*, the genes encoding the GM-CSF-Rα and GM-CSF-Rβ chains (63).

Increased circulating levels of GM-CSF have been recently described in patients with COVID-19 compared to healthy controls (4). A paper from China appearing on the preprint online platform bioRxiv reported that in patients with COVID-19, especially those admitted to the ICU, CD4^+^ T lymphocytes were rapidly activated in the lung to pathogenic T helper (Th) 1 cells and generated GM-CSF and IL-6. This potent pro-inflammatory environment strongly induced CD14^+^CD16^+^ monocytes, which also released GM-CSF and IL-6, further worsening the cytokine storm. These aberrant and numerous GM-CSF^+^-IL-6^+^ cells may enter the lungs and explain the detrimental actions provided by hyperinflammation in the most severe and even fatal cases (64).

## GM-CSF as a Therapeutic Strategy in COVID-19 Pneumonia

In light of the results in animal studies following the intranasal administration of GM-CSF in the setting of respiratory infections, two human recombinant GM-CSF (hrGM-CSF), sargramostim and molgramostim, were investigated in humans (65–67). Sargramostim was tested in a randomized, double-blind, placebo-controlled clinical trial in patients with acute lung injury/ARDS (67). The drug was administered as an intravenous infusion once daily for 14 days at a dosage of 250 μg/m^2^. The study showed no significant difference in the number of ventilator-free days, organ failure-free days, and 28-day mortality between the hrGM-CSF and placebo groups; there was also no difference in the number of serious adverse events (67). A randomized, double-blind, placebo-controlled phase II study tested the effects of low-dose hrGM-CSF (molgramostim, 3 μg/kg daily) for 5 days in patients in addition to the standard of care in critically ill patients with severe sepsis and respiratory dysfunction (65). The study found that hrGM-CSF was associated with an improvement in gas exchange and functional activation of pulmonary macrophages; however, there was no improvement in 30-day survival (65). In another randomized, double-blind, placebo-controlled clinical trial in patients with bacterial and fungal abdominal sepsis, molgramostim 3 μg/kg daily for 4 days was administered in addition to standard of care. The treatment group had a reduction in the rate of infectious complications and in the length of hospitalization (66).

In the early phases of viral infections, GM-CSF's role may be protective as it helps limit virus-related injury. For this reason, an inhaled formulation of sargramostim is being tested in patients with COVID-19-related acute hypoxic respiratory failure (NCT04326920).

## Inhibition of GM-CSF Signaling in COVID-19 Pneumonia

In later stages of COVID-19, the severity of the illness appears to be driven by the inappropriate release of several cytokines, such as IL-6 and GM-CSF. These mediators are involved in the inflammatory lung injury, predisposing patients to respiratory failure and eventually ARDS. Therefore, inhibition of GM-CSF signaling may be a reasonable treatment in this stage of disease. This is supported by pre-clinical data in CRS showing that GM-CSF blockade reduced CAR T-cell therapy-related toxicity by preventing CRS development without affecting its therapeutic activity (68).

Mavrilimumab is a high-affinity monoclonal IgG4 antibody against GM-CSF-Rα [(69); Figure 1C]. The efficacy and safety of mavrilimumab have been studied in rheumatoid arthritis (RA) and showed promising results. In a phase 2b multicenter placebo-controlled study, patients with moderate-to-severe RA were randomized to receive different dose levels of mavrilimumab (30, 100, and 150 mg subcutaneously every 2 weeks) or placebo. Mavrilimumab at a dose of 150 mg subcutaneously every 2 weeks was the most effective in improving clinical and laboratory disease activity (70). No substantial differences in adverse events or severe adverse events were observed between groups (70). These results on safety and efficacy were confirmed in a phase 2 double-blind randomized trial evaluating the use of mavrilimumab at a dose of 100 mg subcutaneously every other week in long-standing RA patients (71). A *post-hoc* analysis of these studies has shown that the administration of mavrilimumab was associated with a significant downregulation of the macrophage-derived chemokine C-C motif chemokine ligand 22 (CCL22) and IL-6 (72), related to a direct inhibition of the proinflammatory cytokine release from myeloid cells. Mavrilimumab also showed a decreased expression of IL-22/IL-17-associated transcripts, the latter suggesting an indirect suppressive effect of mavrilimumab on T cell activation (72). Moreover, a sustained suppression of serum markers of disease activity, such as C-reactive protein and IL-6, was observed in RA patients treated with mavrilimumab (73). Mavrilimumab is currently under investigation for the treatment of giant cell arteritis (NCT03827018).

A prospective interventional single-center cohort study tested the efficacy and safety of mavrilimumab in patients with severe COVID-19 pneumonia and evidence of hyperinflammation in Italy (74). Thirteen non-mechanically ventilated patients with severe COVID-19 pneumonia and hyperinflammation were treated with a single intravenous dose of mavrilimumab 6 mg/kg upon admission to the hospital. Twenty-six non-mechanically ventilated patients with severe COVID-19 pneumonia and hyperinflammation and with similar baseline characteristics were evaluated as a control-group. All patients received similar standard of care therapy, including antivirals and antibiotics. Over the course of the 28-day follow-up period, mavrilimumab-treated patients experienced earlier and improved clinical outcomes than control-group patients, including earlier weaning from supplemental oxygen and shorter hospitalizations. Death occurred in 0% (*n* = 0/13) of mavrilimumab-treated patients by day 28 compared to 27% (*n* = 7/26) of control-group patients (74). These data are consistent with the hypothesis that excessive host immune response driven by T cells and monocytes may have a central role in the pathogenesis of COVID-19 pneumonia. A randomized controlled trial is being designed and is now active (Mavrilimumab in Severe COVID-19 Pneumonia and Hyper-inflammation [COMBAT-19], NCT04397497).

Five monoclonal antibodies targeting GM-CSF (gimsilumab, otilimab, namilumab, lenzilumab, and TJ003234) are in development and are currently under investigation mainly for the treatment of RA. The principal clinical trials both completed and ongoing are described in Table 1 (75, 78). Recently, TJ003234 (also known as TJM2) obtained the US Food and Drug Administration (FDA) clearance to start a clinical study for COVID-19 associated CRS (I-Mab)^1^. Additionally, lenzilumab has received FDA approval for compassionate use in COVID-19 patients (FDA)^2^, while a phase 3 study is ongoing. A clinical trial has also been approved for gimsilumab for the treatment of COVID-19 and is now enrolling patients in the US (NCT04351243) (Figure 1C). In addition, CSL311 is a monoclonal antibody targeting the GM-CSF-Rβ, common to GM-CSF, IL-3, and IL-5. A phase 1 trial is evaluating the safety and tolerability of this drug in patients with asthma (Table 1).

**Table 1:** Clinical trials on currently available GM-CSF blockers.

**Antibody**	**Study design**	**Dose**	**Patients**	**Results**	**References**
**Gimsilumab** (MORAb-022) human anti-GM-CSF IgG1 MA	Phase 1 randomized, double-blind, placebo-controlled, single-dose, dose-escalation (NCT01357759)	Intravenous infusion at increasing doses of 0.36, 0.7, 1, 3, or 10 mg/kg	25 patients with mild to moderate RA and 26 healthy subjects	DAS28-CRP score decreased according to dose regimen ACR improvement with the 10mg/kg dose Drug well-tolerated	(75)
**Gimsilumab** (MORAb-022, KIN1901) human anti-GM-CSF IgG1 MA	Phase 1 randomized, double-blind, placebo-controlled	Escalating single-dose or once-weekly repeat-dose SC	36 patients, 4 cohorts of healthy subjects and 1 cohort of patients with ankylosing spondylitis	Ongoing Aim: to assess the efficacy of gimsilumab in the treatment of ankylosing spondylitis compared to placebo	NCT04205851
**Otilimab** (GSK3196165 MOR103) human high-affinity anti-GM-CSF IgG1 MA	Phase 2a double-blind, placebo-controlled, parallel group (NCT02799472)	SC injection of 180 mg weekly for 5 weeks and then every other week until week 10	39 subjects with active RA	Reduction of synovial inflammation at week 12 180 mg dose was the most effective AE similar in the two groups, and no SAE observed	(76)
**Otilimab** (GSK3196165 MOR103) human high-affinity anti-GM-CSF IgG1 MA	Phase 2b double-blind, placebo-controlled, dose-adaptive (NCT02504671)	22.5, 45, 90, 135, or 180 mg SC weekly for 5 injections, then every other week until week 50	222 patients with active moderate-to-severe RA	Otilimab 180 mg improved DAS28-CRP, ACR20 response, VAS pain, and patient global assessment AE similar across treatment groups; no drug-related SAEs	(77)
**Otilimab** (GSK3196165 MOR103) human high-affinity anti-GM-CSF IgG1 MA	Phase 3 randomized, multicenter, double-blind	150 mg SC weekly or 90 mg SC weekly, both with methotrexate	Estimated enrollment: 1,500 patients with moderate-to-severe active RA with inadequate response to methotrexate	Recruiting Aim: to assess the safety and efficacy of otilimab in combination with methotrexate compared to placebo and tofacitinib	NCT03980483
**Otilimab** (GSK3196165 MOR103) human high-affinity anti-GM-CSF IgG1 MA	Phase 3 randomized, multicenter, double-blind	150 mg SC weekly or 90 mg SC weekly, both with DMARD(s)	Estimated enrollment: 1,500 patients with moderate-to-severe active RA with inadequate response to DMARD(s)	Recruiting Aim: to assess the safety and efficacy of otilimab in combination with DMARD(s) compared to placebo and tofacitinib	NCT03970837
**Otilimab** (GSK3196165 MOR103) human high-affinity anti-GM-CSF IgG1 MA	Phase 3 randomized, multicenter, double-blind study	150 mg SC weekly or 90 mg SC weekly, both with DMARD(s)	Estimated enrollment: 525 patients with moderate-to-severe active RA with inadequate response to DMARD(s) or JAK inhibitors	Recruiting Aim: to assess the safety and efficacy of otilimab in combination with DMARD(s) compared to placebo and sarilumab	NCT04134728
**Namilumab** (AMG203) human high-affinity anti-GM-CSF IgG1 MA	Phase 2 randomized, double-blind, placebo-controlled (NCT02379091)	20, 80, or 150 mg SC with methotrexate	108 patients with RA with no response to methotrexate or TNF inhibitors	Dose-response effect observed. DAS28-CRP was mostly improved in the 150 mg group Similar incidence of SAEs among different doses. URIs were the most frequent AE	(78)
**Namilumab** (AMG203) human high-affinity anti-GM-CSF IgG1 MA	Phase 2 proof-of-concept, multicenter, randomized, double-blind, placebo-controlled	40, 100, 160, or 300 mg SC	122 patients with moderate-to-severe plaque psoriasis	No statistical difference in efficacy between placebo and namilumab groups	NCT02129777
**Namilumab** (AMG203) human high-affinity anti-GM-CSF IgG1 MA	Phase 2a proof-of-concept, randomized, double-blind, placebo-controlled	150 mg SC	42 patients with axial spondyloarthritis	Ongoing Aim: to assess efficacy of namilumab in the treatment of axial spondyloarthritis compared to placebo	NCT036226589
**Lenzilumab** (KB003) recombinant high-affinity anti-GM-CSF IgG1 MA	Phase 2 randomized, placebo-controlled, dose-ranging	70, 200, or 600 mg IV infusion	9 patients with RA with inadequate response to biologic therapy	Study terminated due to a refocus of the program development	NCT00995449
**Lenzilumab** (KB003) recombinant high-affinity anti-GM-CSF IgG1 MA	Phase 1 multicenter open-label, repeat-dose, dose-escalation	200, 400, or 600 mg IV infusion once monthly for a 28-day dosing cycle	15 subjects with previously treated CMML	Completed No results available to date Aim: to examine the safety and determine the recommended Phase 2 dose of Lenzilumab in patients with CMML	NCT02546284
**TJ003234 (TJM2)** recombinant humanized anti-GM-CSF IgG1 MA	Phase 1 randomized double-blind, placebo-controlled, single ascending doses	0.3, 1, 3, or 10 mg/kg via single IV infusion	32 healthy subjects	Completed No results available Aim: to determine safety, tolerability and the MTD	NCT03794180
**CSL311** Human β common receptor for GM-CSF, IL-3, and IL-5 antagonist MA	Phase 1 randomized, double-blind, placebo-controlled, single ascending and multiple ascending doses	Single ascending and multiple ascending dose	74 patients with mild asthma	Recruiting Aim: to assess the safety and tolerability of CSL311	NCT04082754

*ACR, American College of Rheumatology score; AE, adverse event; CMML, Chronic Myelomonocytic Leukemia; DAS28-CRP, disease activity score-28 with CRP; DMARD(s), disease-modifying antirheumatic drugs; IL, interleukin; IV, intravenous; JAK, janus kinase; MA, monoclonal antibody; MTD, maximum dose tolerated; RA, rheumatoid arthritis; SAE, severe adverse event; SC, subcutaneous; TNF, tumor necrosis factor; URIs, upper respiratory infections; VAS, visual analog scale*.

Because GM-CSF is a key mediator in pulmonary homeostasis, there is the theoretical concern that inhibition of GM-CSF signaling by either binding to GM-CSF or blocking the receptor may result in dysfunctional AMs, leading to PAP and development of new infections. Fortunately, there has yet to be a case of PAP reported with the use of anti-GM-CSF monoclonal antibodies. This may be due to the fact that patients with autoimmune PAP have to reach a “critical threshold” of neutralizing antibodies to develop the disease, and the doses currently being utilized in clinical trials may not reach this threshold (79). This may actually be true for the chronic use where the low level of lung penetration of the 100–150 mg subcutaneously every 2 weeks may not provide the level of necessary inhibition (80). However, in the case of COVID-19 pneumonia and hyperinflammation, the lung penetration of the drug may be critical. This is the reason why the dose has been increased from 1.5–2 mg/kg subcutaneously to 6–10 mg/kg intravenously. This means that PAP should not necessarily be an issue in the COVID-19 treatment in that a single intravenous dose is being given and it will wear off in a month, while PAP is a disease caused by chronic inhibition over years.

## Conclusions

As COVID-19 pneumonia is likely to be aggravated by a cytokine storm, immunomodulation gained importance as a possible therapeutic strategy to this disease. A wealth of IL-6 and IL-1 blockade trials are ongoing and results are awaited. However, an approach targeting hyperinflammation upstream of IL-1 and IL-6 as well as neutrophils and macrophages may be envisioned through GM-CSF signaling. GM-CSF is an immunomodulatory cytokine that may help to clear respiratory microbes by stimulating AMs. A clinical trial with a hrGM-CSF, sargramostim, will be conducted in COVID-19 patients with the rationale that it may help clear the SARS-CoV-2 earlier in the disease course. However, in the later phase of COVID-19 lung injury, the marked elevation in GM-CSF levels as part of the cytokine storm during the onset of COVID-19 pneumonia suggests that GM-CSF may actually be deleterious at this stage of the disease. Blocking GM-CSF signaling could therefore be an effective therapeutic strategy by reducing the cytokine storm, which leads to the progression of acute respiratory failure in patients with hyperinflammation. Multiple clinical trials with inhibition of the GM-CSF pathway are either ongoing or under development.

## Author Contributions

AB, AV, and AA conceived the manuscript. AB and AV drafted manuscript, figure, and tables. TW, EL, PC, BC, PR, KH, LK, BV, LD, and AA critically revised the manuscript. All authors approved the final version.

## Conflict of Interest

AB and AV received a travel grant from Kiniksa Pharmaceuticals Ltd. to attend the 2019 AHA Scientific Sessions (Philadelphia, PA, USA). TW was on a research advisory board for GSK. PC was on a scientific advisory board for Kiniksa Pharmaceuticals. AA has served as a consultant to Kiniksa Pharmaceuticals Ltd. The remaining authors declare that the research was conducted in the absence of any commercial or financial relationships that could be construed as a potential conflict of interest.

## Footnotes

^1^I*-Mab Announces IND Clearance from FDA for TJM2 to Treat Cytokine Release Syndrome (CRS) Associated with Severe Coronavirus Disease 19 (COVID-19)*. Available online at: https://www.biospace.com/article/releases/i-mab-announces-ind-clearance-from-fda-for-tjm2-to-treat-cytokine-release-syndrome-crs-associated-with-severe-coronavirus-disease-19-covid-19-/ (accessed April 11, 2020).

^2^*FDA Approves Emergency IND Use of Humanigen's Lenzilumab for Compassionate Use in COVID-19 Patients*. Available online at: https://www.humanigen.com/press/FDA-Approves-Emergency-IND-Use-of-Humanigen%E2%80%99s-Lenzilumab-for-Compassionate-Use-in-COVID-19-Patients (accessed April 11, 2020).
