# Supplementary material for: Targeting GM-CSF in COVID-19 Pneumonia: Rationale and Strategies
Source: Front Immunol. 2020 Jul 3;11:1625. doi: 10.3389/fimmu.2020.01625 (PMC7348297; doi:10.3389/fimmu.2020.01625)
Supplement: Supplementary file 1 [file Table_1.docx]

**Supplementary Table 1. Studies that investigated GM-CSF, IL-6, and ferritin in SARS-CoV-2 pneumonia**

| **Authors** | **Patients** | **Biomarkers** | **Results** |
| --- | --- | --- | --- |
| Huang *et al.* (Huang et al., 2020) | 41 patients | IL-6 and GM-CSF | Higher levels of IL-6 and GM-CSF were found in patients admitted to ICU compared with healthy controls (p=0.003 and p=0.002, respectively); however, there was no difference in the levels between patients with COVID-19 admitted to ICU and not admitted to ICU. GM-CSF was higher in patients not admitted to ICU compared with healthy controls (p=0.002). No difference was found for IL-6 levels between healthy controls and patients with COVID-19 not admitted to ICU. |
| Yuan *et al.*  (Yuan et al., 2020) | 94 patients | IL-6 | Higher levels of IL-6 were recorded in patients with severe compared to moderate disease until day 9. On day 3, IL-6 levels of patients with moderate and severe disease peaked (p<0.01 for severe vs. moderate disease), which then dropped down on day 6 and further on day 12. |
| Gao *et al.*  (Gao et al., 2020) | 43 patients | IL-6 | IL-6 was greatly increased in patients with severe COVID-19 compared to those with mild disease (36 [23.0-59.2] vs. 10.6 [5.1-24.2] pg/mL; p=0.002). IL-6 >24.3 pg/mL was found to independently predict COVID-19 severity (OR 17.3, 95%CI 2.4-123.9; p=0.005). |
| Bo *et al.*  (Bo, 2020) | 20 patients | Ferritin | At baseline, ferritin was higher in very severe compared to severe COVID-19 patients (291.1 [102.1- 648.4] vs. 1006.2 [408.3- 1988.2] ng/mL, p<0.05). |
| Ji *et al.*  (Ji, 2020) | 49 patients | Ferritin | On admission, ferritin was 502.1±444.8 ng/mL, with elevated levels among patients progressing to severe disease (318.1±257.8 vs. 907.4 ± 593.7 ng/mL; p<0.001).  Ferritin >400 ng/mL increased the risk of progression to severe disease (HR: 7.1, 95% CI: 2.6-19.8, p=0.002). Ferritin >400 ng/mL was included in a 4-point score along with comorbidities, age >50, and lymphocyte count <1,500/μL. The presence of 3-4 factors significantly increased the risk of progression to severe disease, intubation, or ICU admission compared with 0-2 risk factors (OR 6.2, 95 % CI: 1.7-22.8, p=0.006). |
| Ruan *et al.*  (Ruan et al., 2020) | 150 patients | IL-6 and ferritin | IL-6 was nearly doubled in dead patients compared with discharged ones (11.4 ±8.5 vs. 6.8±3.61; p<0.001); the same was observed for ferritin (1297.6±1030.9 vs. 614.0±752.2; p<0.001). |
| Chen *et al.*  (Chen et al., 2020) | 99 patients | IL-6 and ferritin | On admission, both IL-6 and ferritin were elevated (7.9 [6.1-10.6] pg/mL and 808.7±490.7 ng/mL, respectively). |
| Wu *et al.*  (Wu et al., 2020) | 201 patients | IL-6 and ferritin | Baseline levels of IL-6 and ferritin were elevated (6.98 [5.46-9.02] pg/mL and 594.0 [315.7-1266.2] pg/mL, respectively).  Significantly higher levels of IL-6 were found in patients with ARDS compared with those without (difference 0.93 pg/mL, 95% CI 0.1-2.0 pg/mL; p=0.03). The same was true for ferritin levels (difference 545.50 ng/mL, 95% CI 332.1-754.4 ng/mL; p<0.01). In patients with ARDS, non-survivors showed significantly higher levels of IL-6 (difference 3.88 pg/mL, 95% CI 2.2-6.1 pg/mL; p<0.001) but not of ferritin (difference 102.55 ng/mL, 95% CI −185.63-412.71 ng/mL; p=0.34) compared to survivors.  IL-6 did not predict the development of ARDS (1.02 [1.00-1.05]; p=0.09), but did increase the risk of progression from ARDS to death (1.03 [1.01-1.05]; p=0.01). Ferritin (>300 vs ≤300 pg/mL) predicted the development of ARDS (HR 3.5, 95% CI 1.5-8.2; p=0.003) but failed to predict the progression from ARDS to death (HR 5.3, 95% CI 0.7-38.5; p=0.10). |
| Liu *et al.*  (Liu et al., 2020) | 80 patients with severe disease | IL-6 and ferritin | On admission, ferritin was 690.20±864.3 μg/L, with higher levels in severe compared to non-severe patients (827.2±916.9 vs. 155.70±187.3 μg/L, p=0.001). A similar trend was observed for IL-6 (p=0.016).  Increased IL-6 levels at baseline positively correlated with bilateral, interstitial lung involvement (r=0.453, p=0.001), body temperature (r=0.521, p<0.001), ferritin (r=0.606, p=0.001), high-flow oxygen therapy (r=0.251, p=0.007), and mechanical ventilation (r=0.223, p=0.017). |
| Zhou *et al.*  (Zhou et al., 2020a) | 191 patients | IL-6 and ferritin | IL-6 was higher in non-survivors than in survivors (11.0 [7.5-14.4] vs. 6.3 [5.0-7.9] pg/mL; p<0.001); the same was true for ferritin (1,435.3 [728.9-2,000] vs. 503.2 [264.0-921.5] μg/L; p<0.001).  IL-6 progressively increased from day 4 to day 19 in non-survivors (9.5 🡪 26.4 pg/mL), while IL-6 remained low (around 6 pg/mL) in survivors across the same period. IL-6 was significantly higher in survivors compared to non-survivors at all time points but on day 4 (p<0.05).  Ferritin increased in survivors compared to non-survivors from day 4 to day 19 (1,025 🡪 >2,000 μg/L vs. 393 🡪 432 μg/L, p<0.05). |

All patients have been diagnosed COVID-19 using real-time polymerase chain reaction.

For most of the studies, normal values for IL-6 and ferritin were 0-7 pg/mL and 21.0-274.7 ng/mL, respectively.

Abbreviations. ARDS: acute respiratory distress syndrome. CI: confidence interval. COVID-19: Coronavirus disease 2019. HR: hazard ratio. ICU: intensive care unit. IL: interleukin. OR: odds ratio.
